# Porous Optically Transparent Cellulose Acetate Scaffolds for Biomimetic Blood-Brain Barrier*in vitro* Models

**DOI:** 10.3389/fbioe.2021.630063

**Published:** 2021-02-10

**Authors:** Attilio Marino, Micol Baronio, Umberto Buratti, Elisa Mele, Gianni Ciofani

**Affiliations:** ^1^Smart Bio-Interfaces, Istituto Italiano di Tecnologia, Pontedera, Italy; ^2^Department of Mechanical and Aerospace Engineering, Politecnico di Torino, Turin, Italy; ^3^Materials Department, Loughborough University, Loughborough, United Kingdom

**Keywords:** blood-brain barrier, *in vitro* models, biomimetic substrates, electrospinning, vapor-induced phase separation

## Abstract

*In vitro* blood-brain barrier (BBB) models represent an efficient platform to conduct high-throughput quantitative investigations on BBB crossing ability of different drugs. Such models provide a closed system where different fundamental variables can be efficaciously tuned and monitored, and issues related to scarce accessibility of animal brains and ethics can be addressed. In this work, we propose the fabrication of cellulose acetate (CA) porous bio-scaffolds by exploiting both vapor-induced phase separation (VIPS) and electrospinning methods. Parameters of fabrication have been tuned in order to obtain porous and transparent scaffolds suitable for optical/confocal microscopy, where endothelial cell monolayers are allowed to growth thus obtaining biomimetic BBB *in vitro* models. Concerning VIPS-based approach, CA membranes fabricated using 25% H_2_O + 75% EtOH as non-solvent showed submicrometer-scale porosity and an optical transmittance comparable to that one of commercially available poly(ethylene terephthalate) membranes. CA membranes fabricated *via* VIPS have been exploited for obtaining multicellular BBB models through the double seeding of endothelial cells and astrocytes on the two surfaces of the membrane. Electrospun CA substrates, instead, were characterized by micrometer-sized pores, and were unsuitable for double seeding approach and long term studies. However, the potential exploitation of the electrospun CA substrates for modeling blood-brain-tumor barrier and studying cell invasiveness has been speculated. The features of the obtained models have been critically compared and discussed for future applications.

## Introduction

The blood-brain barrier (BBB) is a highly specialized functional structure of the fully differentiated neurovascular system that allows selective regulation of movement of ions, molecules, and cells between the blood and the brain (Zlokovic, 2008). BBB unique selective properties protect brain parenchyma from toxic substances/pathogens, control the immunologic status of the brain, prevent the passage of large molecules and of circulating blood cells that can damage neuronal tissue, maintain water and ions homeostasis, and allow the carrier-mediated transport of glucose and amino acids (Abbott, 2013). The functionality of the BBB is mainly attributed to: (i) the tight junctions among the endothelial cells of the vases, which remarkably limit the paracellular non-specific crossing of substances through the barrier (Bauer and Traweger, 2016), (ii) the basement membrane, a non-cellular component consisting of extracellular matrix proteins which provides support to the cells of the neurovascular unit and contributes to the integrity of the barrier (Xu et al., 2019), (iii) astrocytes, which play a role in maintaining BBB integrity and may represent a supplementary physical barrier with astrocytic endfeet (Kubotera et al., 2019), (iv) pericytes, which regulate capillary blood flow and permeability of the BBB through paracrine signaling (Liu et al., 2012), and (v) microglia, the role of which in modulating BBB integrity remains unclear (Haruwaka et al., 2019).

Disruption of the BBB can be associated to inherited human monogenic neurological diseases affecting individual cell types involved in BBB structure, function, and development (Zhao et al., 2015). Moreover, deficits of BBB functionality have been also reported in other pathologic conditions, such as, for example, brain cancer, Parkinson’s disease, Alzheimer’s disease, stroke, diabetes, and multiple sclerosis (Shimizu et al., 2018). The dynamics of BBB in physio-pathological conditions, the different biochemical mechanisms involved in BBB crossing, and the delivery of drugs to the brain through BBB are objects of intensive research in biomedicine and nanomedicine for the development of new therapies for brain cancer and neurodegenerative diseases (Dong, 2018).

Although several new drugs/compounds showed great potential for the treatment of brain diseases, most of them could not be successively exploited in clinics because resulted unable to efficiently cross the BBB. *In vitro* BBB models, from static biomimetic 2D models (Bischel et al., 2016), to 2D microfluidic systems,(De Pasquale et al., 2020) up to considering the first examples of 3D real-scale fluidic systems (Marino et al., 2018), represent an efficient platform to conduct high-throughput quantitative investigations of drug delivery to the brain. *In vitro* BBB models provide a closed system where different fundamental variables (e.g., drug concentration, pH, temperature, and permeability) can be easily tuned and monitored, thus providing precious and detailed information about the BBB crossing in real time and at cellular/subcellular level (Jackson et al., 2019). Finally, drug screening on *in vitro* BBB models reduces the number of investigations on animal models (which, in any case, remain necessary preclinical validations) and, therefore, overcomes issues related to the scarce accessibility of the brain tissue, and limits important ethical concerns. In this context, commercially available porous membranes made of polycarbonate, poly(ethylene terephthalate) or poly(tetrafluoroethylene) have been exploited not only for *in vitro* BBB modeling, yet also for studies regarding other biological barriers (e.g., intestinal epithelial barrier) and cell invasion assays. However, materials used for these commercially available membranes do not accurately mimic human tissue and, with respect to the native basement membrane, are thicker and less porous (Bischel et al., 2016). For this reason, it is necessary to explore alternative materials and fabrication approaches to obtain and commercialize innovative biomimetic reproducible systems (Qi et al., 2018).

In this work, we propose the fabrication of porous bio-scaffolds of cellulose acetate (CA) suitable for optical/confocal microscopy, cell culture, and development of endothelial cell monolayers for the set-up and characterization of biomimetic BBB *in vitro* models. CA is the acetate ester of cellulose, widely used in biotechnology for immobilization of biomolecules, tissue engineering, biosensing, nutraceutical delivery, bioremediation, and development of antimicrobial scaffolds (Konwarh et al., 2013; Mohite and Patil, 2014; Courtenay et al., 2018). Cellulose-based bio-scaffolds are biologically inert long-lasting non-enzymatically degradable structures that favor cell adhesion and proliferation (Hickey and Pelling, 2019). Vapor-induced phase separation (VIPS) (Ismail et al., 2020) and electrospinning (Soares et al., 2018) approaches have been selected and optimized for the fabrication of the biomimetic CA scaffolds and, subsequently, BBB models have been developed and characterized on selected porous and transparent scaffolds. The properties of the obtained models have been finally compared and discussed for future applications.

## Results

### Preparation and Characterization of Porous CA Membranes by VIPS

The VIPS process was used to create transparent and porous CA scaffolds by controlling polymer precipitation and solvent evaporation from polymer-solvent mixtures exposed to an atmosphere of non-solvent vapors. Three different CA solutions (7.5, 10.0, and 15.0% wt CA in acetone) and five different non-solvent H_2_O-EtOH mixtures (100% H_2_O, 75% H_2_O + 25% EtOH, 50% H_2_O + 50% EtOH, 25% H_2_O + 75% EtOH, and 100% EtOH) were used. The CA-acetone solutions were deposited on glass slides to form uniform wet coatings, which were exposed to the gaseous non-solvents. This induced thermodynamic instabilities in the wet films, leading to solid-liquid demixing and formation of polymer-rich and polymer-lean regions. The complete evaporation of the solvent determined the final structure (porosity) and performances (optically and mechanically) of the CA membranes. Regardless of the non-solvent used, the membranes produced from 15.0% wt CA solutions were remarkably opaque; those produced from 7.5% wt CA solutions were inhomogeneous and several cases of membrane wrinkling/cracking were observed; those derived from 10.0% wt CA solutions instead showed improved light transparency and mechanical stability (Supplementary Figure 1). Hence, these membranes (10% wt CA solutions) were selected for further imaging and biological characterization.

**FIGURE 1:**
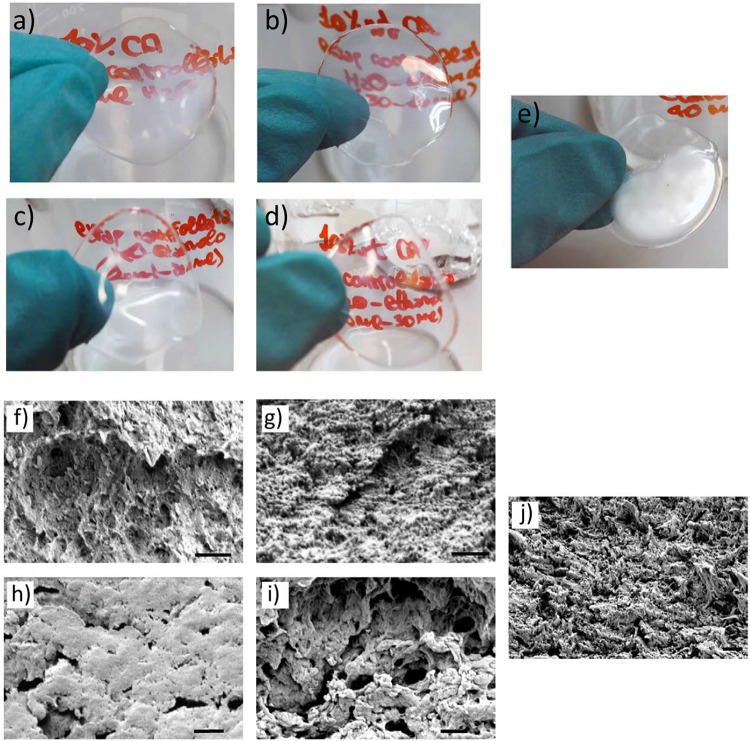
CA membranes (from 10.0% wt CA solutions) prepared *via* VIPS. **(A–E)** Photographs and **(F–J)** SEM images of the transverse section of CA membranes obtained with different non-solvent mixtures: **(A,F)** 100% H_2_O, **(B,G)** 75% H_2_O + 25% EtOH, **(C,H)** 50% H_2_O + 50% EtOH, **(D,I)** 25% H_2_O + 75% EtOH, and **(E–J)** 100% EtOH. Scale bar: 1 μm.

In Figure 1, the photographs (Figures 1A–E) and the scanning electron microscopy (SEM) images (Figures 1F–J) of CA membranes obtained with different non-solvents are shown. Qualitative observations on the different CA membranes have been performed prior to select and characterize the best candidate. Best transparency was observed in samples prepared with 50% H_2_O + 50% EtOH (Figure 1C) and 25% H_2_O + 75% EtOH (Figure 1D) non-solvents. A decreased transparency was observed when using 100% H_2_O (Figure 1A) and 75% H_2_O + 25% EtOH (Figure 1B); CA membranes prepared by using 100% EtOH as non-solvent (Figure 1E) resulted extremely opaque and optically heterogeneous. SEM imaging of the CA membrane transverse sections are reported in Figures 1F–J and shows as samples obtained with 50% H_2_O + 50% EtOH (Figure 1H) non-solvents were characterized by a very scarce micro/nanoporosity with respect to the other samples; only few cracks could be observed in the transverse section of this sample. All the other samples displayed a remarkable membrane porosity (Figures 1F,G,I,J), especially the sample obtained by using 25% H_2_O + 75% EtOH non-solvent (Figure 1I). The main qualitative observations on CA membranes regarding optical transparency, porosity, mechanical integrity, and fabrication reproducibility are summarized in Table 1. This was used to define a protocol, while quantitative evaluations have been subsequently performed on selected scaffolds. Considering the aforementioned properties, CA membranes fabricated by using 25% H_2_O + 75% EtOH non-solvent represented the best candidates and have been selected for developing BBB models.

**TABLE 1:** Optical transparency, porosity, mechanical integrity, and fabrication reproducibility of CA membranes obtained *via* vapor-induced phase separation (VIPS) using different non-solvent mixtures.

**Non-solvent**	**Transparency**	**Porosity**	**Stability**	**Reproducibility**
H_2_O (100%)	+	++	+	++
H_2_O (75%) + EtOH (25%)	++	+++	++	−
H_2_O (50%) + EtOH (50%)	+++	−	−	+
H_2_O (25%) + EtOH (75%)	+++	+++	+++	+++
EtOH (100%)	−	+++	+++	−

*+++, optimal: ++, good; +, moderate; −, scarce.*

### Preparation of BBB Models With Selected CA Membranes

**FIGURE 2:**
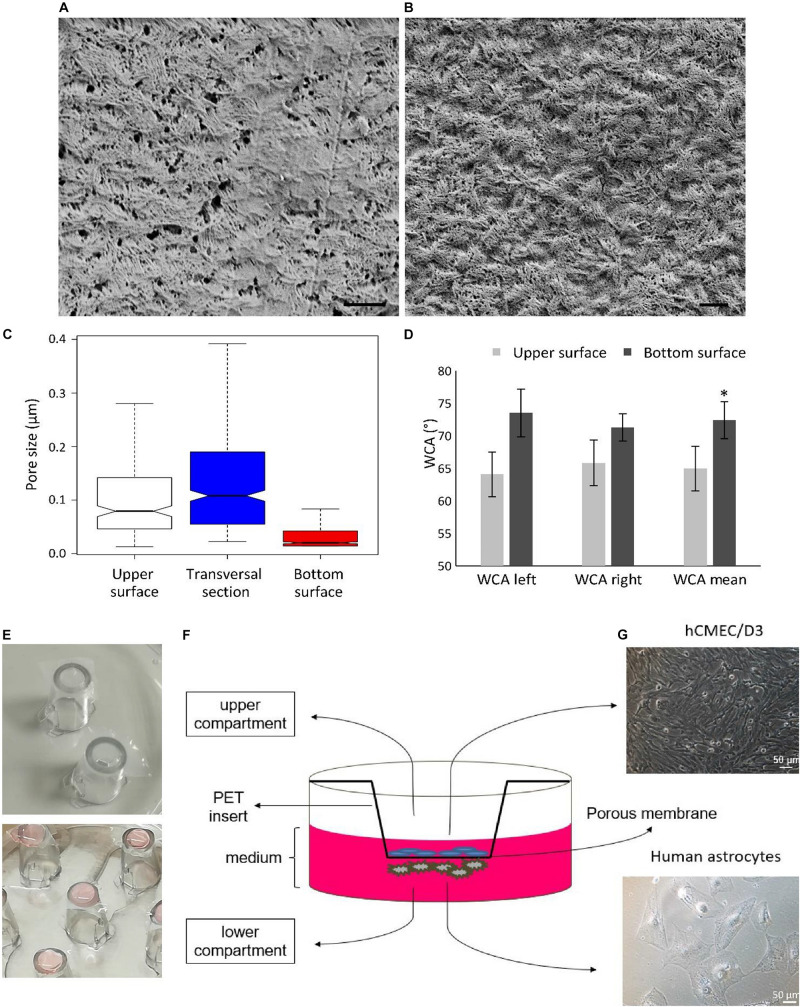
Characterization of the CA membranes fabricated by using 25% H_2_O + 75% EtOH non-solvent and preparation of the BBB model. Representative SEM scans of the **(A)** upper and **(B)** bottom surfaces. Scale bar: 1 μm. **(C)** Pore size distributions of the upper, bottom, and transversal surfaces. **(D)** Water contact angles (WCA) of the bottom and upper surfaces; WCA was measured on the left and right interfaces between the water drop and the substrate; the mean WCA value was calculated from the left and right angles. **(E)** Representative images showing the insert preparation with CA membranes (top), and the cell seeding of human astrocytes on the bottom surface of the membranes (bottom). **(F)** Scheme of the multicellular *in vitro* BBB model with **(G)** hCMEC/D3 human cerebral microvascular endothelial cells seeded at high confluence on the upper surface (top) and human astrocytes on the bottom surface of the CA membranes. *p < 0.05.

Further characterization on the CA membranes fabricated using 25% H_2_O + 75% EtOH non-solvent (hereafter, for simplicity, they will be named as “CA membranes”) and the preparation of the BBB models are shown in Figure 2. Representative SEM scans of the two surfaces (bottom and upper) of the CA membranes are reported in Figures 2A,B, respectively. The pore size distributions without outliers of the two surfaces and of the transversal section are shown in Figure 2C; while the pore size distributions with outliers are shown in Supplementary Figure 2. The bottom surface of the CA membranes, which represents the surface in direct contact to the glass substrate during sample preparation, was characterized by a median pore size (20 ± 2 nm) significantly smaller with respect to both the upper surface (79 ± 10 nm; *p* < 0.05; Kruskal–Wallis test followed by Nemenyi–Damico–Wolfe–Dunn -NDWD- *post hoc* test) and the transversal section (108 ± 10 nm; *p* < 0.05; Kruskal–Wallis test followed by NDWD *post hoc* test), where the upper surface is the surface in direct contact with non-solvent vapors during membrane formation. The transversal section was characterized by a significant number of micrometer-sized and submicrometric pore outliers. The pore density θ*_*S*_* of the bottom surface was 1.1 ± 0.3%, significantly lower compared to that one of the transversal section (3.4 ± 1.1%; *p* < 0.05; *t*-test). Despite the fact that the average pore size in the upper surface was lower than that one measured in the transversal section, the porosity of the upper surface (2.6 ± 1.2%) was non-significantly different from that one of the transversal section (*p* > 0.05; *t*-test), due to the high number of pores in the upper surface. Membrane thickness was assessed as 115 ± 22 μm. Water contact angle (WCA) analysis is reported in Figure 2D. Mean WCA measured on the upper surface was 64.9 ± 3.4°, significantly higher compared with that one measured on the bottom surface (72.4 ± 2.9°; *p* < 0.05; *t*-test); this result shows as both surfaces display hydrophilic properties (WCA < 90°), although a small yet significant increased hydrophilicity characterizes the upper surface. In Figure 2E, the photos of the insert preparation by fixing CA membranes (top), and of the human astrocyte cell seeding on the bottom surface of the membranes (bottom) are depicted. After 15 h from astrocyte seeding, inserts were transferred to a 24-well culture plate and human cerebral microvascular endothelial hCMEC/D3 cells were seeded at high confluence on the upper surface of the CA membranes (Figures 2F,G).

### Characterization of BBB Models on CA Membranes

**FIGURE 3:**
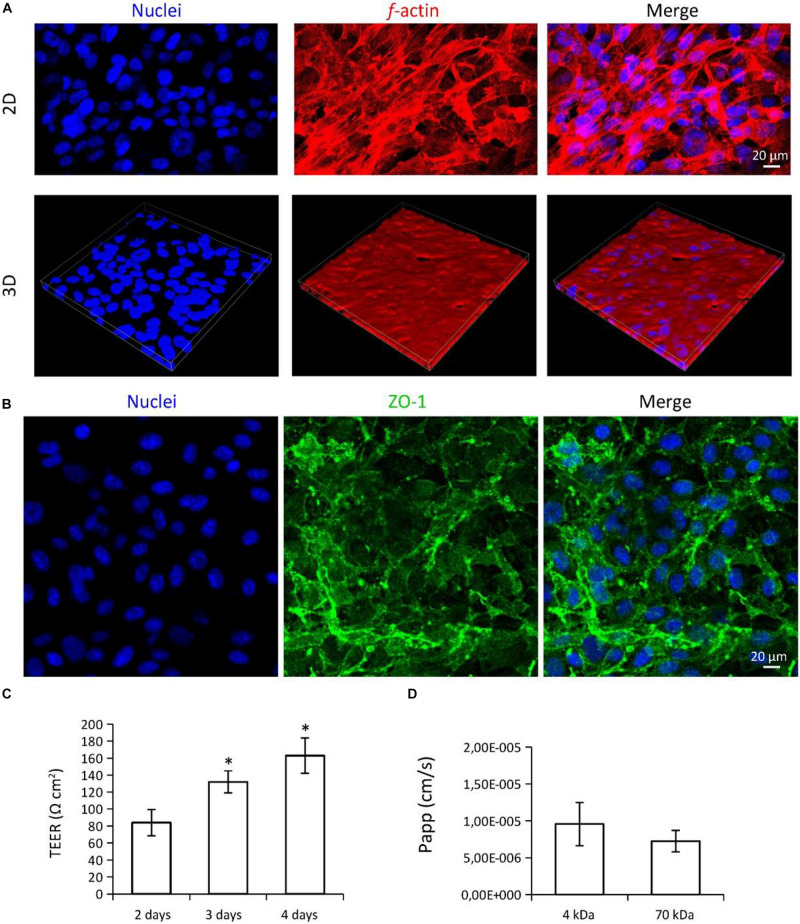
Characterization of the multicellular BBB *in vitro* model. **(A,B)** Confocal laser scanning microscopy (CLSM) imaging of hCMEC/D3 endothelial cells. **(A)** 2D and 3D imaging of *f-*actin (in red) and nuclei (in blue); 3D scan region = 300 μm (*x* axis), 300 μm (*y* axis), 20 μm (*z* axis). **(B)** Immunofluorescence staining and CLSM imaging of ZO-1 tight junction marker (in green), and nuclei (in blue). **(C)** Transendothelial electrical resistance (TEER) measured at different time points (2, 3, and 4 days from seeding) and **(D)** apparent permeability (*P*_*app*_) of the BBB model. ^∗^*p* < 0.05.

The characterization of BBB models is reported in Figure 3. Confocal laser scanning microscopy (CLSM) imaging of the hCMEC/D3 endothelial cells on the CA membranes is shown in Figures 3A,B. Although cell density was not completely homogeneous, endothelial cells almost entirely covered the CA membrane surfaces (Figure 3A; in blue nuclei, in red *f-*actin) and expressed the ZO-1 tight junction marker (Figure 3B; in blue nuclei, in green ZO-1). Transendothelial electrical resistance (TEER) was 84.0 ± 15.4 Ωcm^2^ at day 2, 132.1 ± 12.9 Ωcm^2^ at day 3, and 162.91 ± 20.8 Ωcm^2^ at day 4 (Figure 3C). TEER values at day 4 and at day 3 were both significantly higher than TEER measured at day 2 (*p* < 0.05; ANOVA followed by HSD *post hoc* test). Apparent permeability (*P*_*app*_) of the 4 and 70 kDa FITC-dextrans through the BBB model were 9.55 × 10^–6^ ± 2.95 × 10^–6^ cm/s and 7.24 × 10^–6^ ± 1.45 × 10^–6^ cm/s, respectively (Figure 3D; no significant difference was detected; *p* > 0.05; *t*-test.

### Preparation and Characterization of Electrospun CA Scaffolds

**FIGURE 4:**
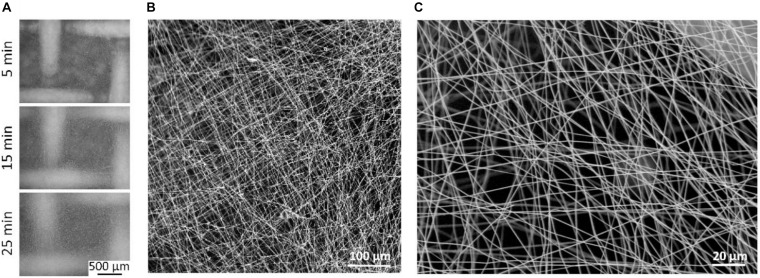
Electrospun substrates by using 15% CA. **(A)** Optical microscope images of the scaffolds prepared on stainless-steel mesh by using three different spinning times (5, 15, and 25 min). **(B)** Low magnification and **(C)** high magnification scanning electron microscopy (SEM) imaging of 25 min electrospun CA scaffolds, as selected candidates for *in vitro* testing.

Cellulose acetate membranes were also prepared using mats of electrospun fibers. Solutions of CA (15, 16, 17, and 18% wt) in acetone were initially tested for electrospinning. The solution at 15% wt CA resulted the most suitable for fibers production, while solutions at CA concentration of 16, 17, and 18% wt were too viscous to be processed by electrospinning. Three different spinning times (5, 15, and 25 min) were tested by using 15% CA solution (Figure 4). The optical microscope images of the scaffolds prepared on stainless-steel mesh at different spinning times are shown in Figure 4A (5 min, top; 15 min, center; 25 min, bottom). Optical transparency, despite improved for shorter spinning times, was maintained in acceptable ranges even in the case of 25 min electrospinning. The structural stability of the mats electrospun for 5 and 15 min was not adequate to support cell growth, and fractures were initiated when detaching, handling, and fixing the mats to the inserts (especially in the case of 5 min spinning time). Therefore, 5 and 10 min electrospun substrates were not exploitable for our scopes. Substrates electrospun for 25 min exhibited both the optical transparency and the structural stability necessary for manipulation, and therefore were selected as candidates for *in vitro* testing. A comparison between optical transmittance of 25 min electrospun CA scaffolds, CA membranes obtained *via* VIPS, and commercially available poly(ethylene terephthalate) (PET) membranes is shown in Supplementary Figure 3. SEM imaging of 25 min electrospun CA scaffolds is shown in Figure 4B (low magnification) and Figure 4C (high magnification). Median pore size and fiber size were, respectively, 1.98 ± 0.05 μm and 587 ± 41 nm.

### Characterization of BBB Models on CA Electrospun Substrates

**FIGURE 5:**
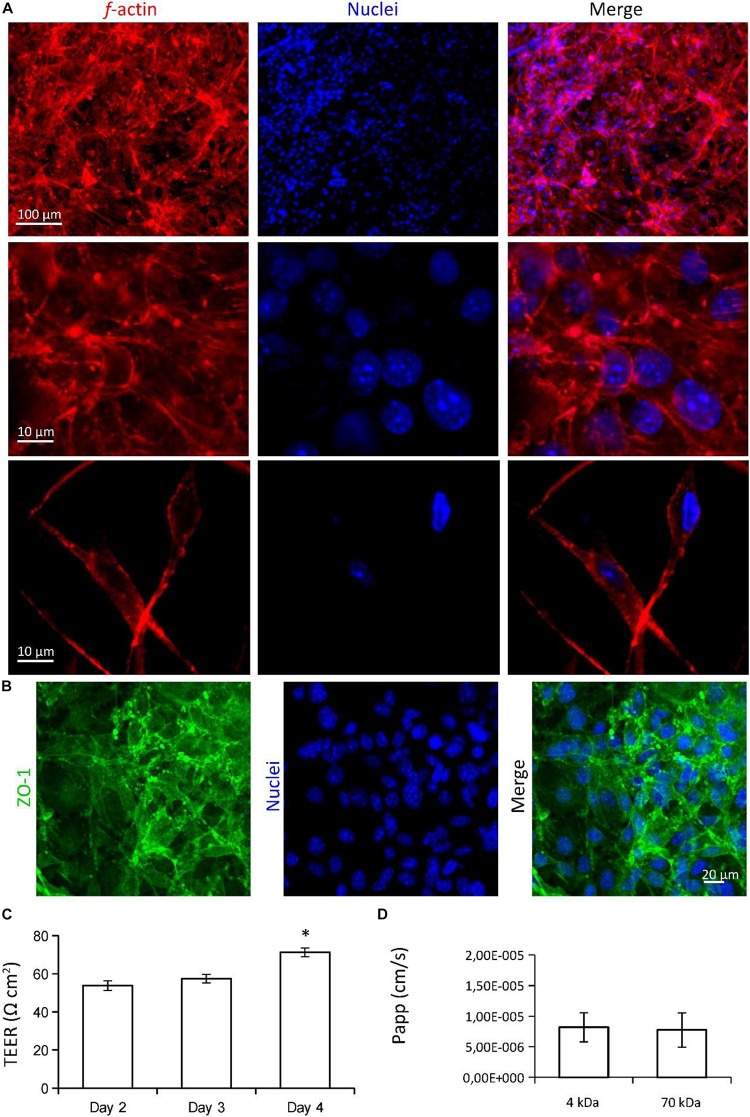
Imaging and characterization of the *in vitro* BBB model on CA electrospun scaffolds (25 min spinning time). **(A)** Confocal laser scanning microscopy (CLSM) imaging of the endothelial cells (*f*-actin in red, nuclei in blue) on the CA electrospun scaffolds. Low magnification (top) and high magnification (middle) imaging show as endothelial cells develop a biological barrier on the surface of the CA scaffold; CLSM imaging of the CA substrate (bottom) shows as few cells were able to migrate inside the scaffold. **(B)** Immunofluorescence staining and CLSM imaging of ZO-1 tight junction marker (in green), and nuclei (in blue). **(C)** TEER measured at different time points (2, 3, and 4 days from seeding). **(D)** Apparent permeability (*Papp*) of the BBB model. *p < 0.05.

Imaging and characterization of the *in vitro* BBB models on CA electrospun scaffolds (25 min spinning time) are presented in Figure 5. Confocal laser scanning microscopy imaging of the endothelial cells (*f*-actin in red, nuclei in blue) on the CA electrospun scaffolds are shown in Figure 5A. Low magnification imaging (top) shows as cells almost entirely cover the surface of the scaffold. High magnification imaging (middle) shows as cells strongly interact each other and develop a biological barrier. Confocal imaging of the CA substrate (bottom) reveals as cells were able to migrate inside the scaffold. Endothelial cells on electrospun substrates expressed the ZO-1 tight junction marker, with an elevated signal localized to the cell-cell-borders (Figure 4B; in blue nuclei, in green ZO-1). TEER values were measured at different time points (2, 3, and 4 days from seeding) and results are plotted in Figure 5C. TEER was 53.7 ± 2.5 Ωcm^2^ at day 2, 57.4 ± 2.2 Ωcm^2^ at day 3, and 71.2 ± 2.3 Ωcm^2^ at day 4, significantly higher with respect to the TEER of the substrate without cells (33.4 ± 3.4 Ωcm^2^; *p* < 0.05; ANOVA followed by HSD *post hoc* test). TEER at day 4 was significantly higher with respect to the TEER at day 2 and day 3 (*p* < 0.05; ANOVA followed by HSD *post hoc* test). Apparent permeability (*P*_*app*_) of the 4 and 70 kDa FITC-dextrans through the BBB model were 8.20 × 10^–6^ ± 2.37 × 10^–6^ cm/s and 7.73 × 10^–6^ ± 2.79 × 10^–6^ cm/s, respectively (Figure 3D; no significant difference was detected; *p* > 0.05; *t*-test).

## Discussion

In this work, CA substrates have been successfully obtained by VIPS and electrospinning. They have been characterized and, for the first time, exploited for developing *in vitro* BBB models. In this section, a comparison between properties and potential use of the two types of CA substrates is reported. In both cases, the main challenge consisted of finding a compromise between structural stability necessary for handling the substrates and optical transparency for performing microscope imaging. Concerning VIPS approach, an important parameter to be calibrated is represented by the solute concentration (in the case of CA solution, the best results were obtained with cellulose 10.0% wt) and the non-solvent used (25% H_2_O + 75% EtOH non-solvent represented the best candidate for optical transparency, porosity, mechanical integrity, and fabrication reproducibility). Regarding electrospinning, CA electrospun substrates with spinning times lower than 25 min were not mechanically stable and easily fractured when handled (i.e., during the substrate detachment from the mesh and the insert assembly); scaffolds electrospun for 25 min exhibited both optimal transparency for microscope imaging and good mechanical stability for substrate manipulation. Interestingly, the optimal parameters used for the electrospinning of the CA solution in our experimental conditions were similar to those chosen by Bischel et al. (2016) for the fabrication of gelatin substrates used as BBB models (15% gelatin solution, 30 min spinning time and 5 μl/min flow rate).

Concerning porosity, CA membranes obtained by VIPS showed high density of nanopores with variable size in the different surfaces, ranging from 20 ± 2 nm in the bottom surface, 79 ± 10 nm in the upper surface, and 108 ± 10 nm in the transversal section. Substrates with bigger pore size were characterized by lower contact angles (i.e., improved wettability), in line with current literature (Chan and Idris, 2012; Chan and Ng, 2018; Zhao et al., 2007). The upper surface has been chosen for cell culturing since the improved wettability is associated with enhanced cell adhesion, which in turn is important for the maintenance of endothelial cell monolayer architecture (Tzoneva et al., 2007). Although the small nanopore size of the CA membranes obtained by VIPS can potentially favor cell adhesion (Huang et al., 2019), this may represent a limit for studying the BBB crossing of certain nanomaterials (i.e., those characterized by a diameter size higher than the pore size). Instead, electrospun CA substrates displayed micrometer size pores (∼2 μm). Similar pore sizes are used in many different commercially available inserts for *in vitro* BBB models or other barrier studies (e.g., from Falcon, Merk, ThermoFisher Scientific), but also in some substrates for cell migration/invasion assay (e.g., from BD Biosciences). Indeed, some cell migration phenomena within the electrospun scaffolds have been observed.

Transendothelial electrical resistance values associated to the CA membranes obtained by VIPS without cells were remarkably higher with respect to those measured in the CA electrospun scaffolds, coherently with the smaller pore size observed. TEER of cell monolayers (after subtracting the substrate resistance) were in line with those estimated by Paradis et al. (2016) on monolayers of human brain microvascular endothelial cells (hBMEC, TEER ranging between 20 and 200 Ω cm^2^). The higher level of TEER of cells on CA membranes obtained by VIPS can be attributed to the easier ability of endothelial cells to cover the nanopores of these substrates with respect to the micropores of the electrospun substrates. However, the crossing of the fluorescent dextran through the *in vitro* BBB model was comparable for the two substrates. Anyway, the detailed comparison of the BBB models obtained with the two methods is not the main scope of the work. Rather, the two types of substrates proved to be suitable for modeling and studying different phenomena. The membranes obtained by VIPS approach are characterized by nanopores and can be used to study the BBB crossing of molecules or small nanostructures (i.e., with a size < 20 nm); while the electrospun substrates can be used for investigating the passage of larger nanostructures and can be also exploited to model the blood-brain-tumor barrier (BBTB), which is characterized by several fenestrations. Moreover, electrospun substrates allow cell migration through the scaffold and may be of interest for invasion assays (which typically last 24–48 h). Instead, the advantages associated with the higher stability of the VIPS CA membranes in biological medium offer the possibility to perform long-term studies and to exploit the double seeding approach for obtaining biomimetic multicellular BBB models.

Concluding, this work represents the first study on the use of CA substrates fabricated by VIPS and electrospinning to obtain BBB *in vitro* models. This investigation demonstrated the possibility of successfully preparing optically transparent and structurally stable substrates with a bioinspired non-degradable material suitable for optical/confocal microscopy, cell culture, and development of endothelial cell monolayers. The potential of electrospun CA substrates for short-term investigation on cancer cell invasiveness will be carefully evaluated in future studies. Specifically, cancer cells will be seeded on the electrospun substrates in multiwell inserts and different cancer cell migration/metastasis inducers will be tested. Taking advantage of the transparency of these scaffolds, confocal imaging will be performed inside the substrates to analyze the migration rate of the cancer cells. Concerning the VIPS-based approach, future works will be devoted to improve and scale up the fabrication process in order to obtain reliable and economical commercial-scale products.

## Materials and Methods

### CA Membrane Preparation and Characterization

Cellulose acetate (Sigma Aldrich, Mn 30 kDa) membranes have been prepared by VIPS. A scheme of the experimental procedure is reported in Supplementary Figure 4. Three different CA solutions with 7.5, 10.0, and 15.0% wt in acetone were prepared. 700 μl of the obtained solutions were dripped on circular glass slides (glass slide diameter = 30 mm). Each glass slide was placed in a beaker and transferred into a sealed container with 40 ml of non-solvent (H_2_O-EtOH mixture) for controlled acetone evaporation. Five different H_2_O-EtOH mixtures have been tested (100% H_2_O, 75% H_2_O + 25% EtOH, 50% H_2_O + 50% EtOH, 25% H_2_O + 75% EtOH, and 100% EtOH) as non-solvents. After 6 days, the containers were opened and the membranes were detached from the glass substrates. Subsequent imaging and characterization were performed on the membranes prepared from solution with 10.0% wt CA due to their superior transparency and mechanical stability performances.

Field emission gun scanning electron microscope (FEGSEM; ZEISS GeminiSEM) imaging has been carried out on Au/Pd-sputtered CA membranes (Quorum Q150R-S sputter; 20 mA current, 90 s deposition time, and argon as plasma gas). SEM imaging was performed on the upper surface, bottom surface, and transversal section of the membranes. For the transversal section imaging, samples were previously immersed in liquid nitrogen (−96°C) for 50 s and subsequently cryofractured.

The pore size analysis has been performed on upper, bottom and transversal surfaces by using ImageJ software^1^. The image analysis has been carried out semi-automatically by imposing a binary threshold and using the default ImageJ selection algorithm, as previously described in other works (George et al., 2018; Kuriakose et al., 2019). The pore size distributions were shown in boxplots by using the MATLAB software. Surface porosity was calculated as ratio between the sum of all the pore areas and the total area of the material, as formulated in Equation 1:

(1) $$\Phi_{S}=\frac{\sum_{i}^{n}{\left(A_{p\,o\,r\,e}\right)}_{i}}{A_{t\,o\,t}}\times 100\%$$

The contact angle measurements were performed by using an Attension^®^ Theta Flex (Biolin Scientific) associated with OneAttension software for live analysis. Specifically, a 5 μl drop of H_2_O was automatically deposited with a hydrophobic dispenser tip on the membrane surface (upper or bottom surface) and WCA determined 10 s after deposition by using a Young–Laplace-fit. WCA was measured at both the left and right interface between the drop and the substrate. For each surface (*n* = 2), three measurements have been performed in three different points of the same sample, and the data expressed as average ± standard deviation.

### *In vitro* Multicellular BBB Models on CA Membranes

A multicellular *in vitro* model of BBB was obtained by using CA-based membranes as porous cell scaffold.

Firstly, commercial poly(ethylene terephthalate) (PET) membranes were removed from cell culture inserts (Falcon) and then substituted with CA membranes; specifically, CA membranes have been fixed into the inserts by depositing a thin ring of poly(dimethylsiloxane) (PDMS; 1:10 dilution of the curing agent in the silicone elastomer curing agent; SYLGARD^TM^) on the upper edge of the insert structure through a syringe tip; PDMS was then dried in the oven at 70°C for 4 h.

Inserts with CA membranes were subsequently used as scaffolds for the co-culture of human astrocytes from healthy brain (derived from cerebral cortex; Innoprot) and of human cerebral microvascular endothelial hCMEC/D3 cells (Biemans et al., 2017) (derived from human temporal lobe microvessels and subsequently immortalized; Merk Millipore). Human astrocytes were cultured in T-75 flasks with high glucose Dulbecco’s Modified Eagle Medium (DMEM; Sigma-Aldrich), supplemented with 1% sodium pyruvate, 1% L-glutamine, 1% non-essential amino acids (Gibco), 100 IU/ml penicillin (Gibco), and 100 μg/ml streptomycin (Gibco); human astrocytes have been seeded on the bottom surface of the CA scaffolds (inserts upside-down) and left in a humid chamber of the incubator to adhere to the membranes (volume seeding 125 μl; seeding density 5 × 10^3^ cells/cm^2^). The surface of a Petri dish was placed on the inserts, in direct contact with the drops of medium used for astrocyte seeding, in order to avoid medium leaking through the membranes due to the surface tension (Supplementary Figure 5). After 15 h, inserts were transferred into a 24-well culture plate and hCMEC/D3 cells were seeded on the upper surface of the CA membranes at a density of 6 × 10^4^ cells/cm^2^.

### Characterization of BBB Models on CA Membranes

The barrier functionality was assessed by analyzing the expression of tight junctions, the TEER and the apparent permeability coefficient *Papp*.

The expression of the tight junctions was assessed by immunofluorescence against *zonula occludens-1* (ZO-1), similarly as previously described (Marino et al., 2019). At day 4 of culture, inserts were dissociated with a blade, cells were fixed for 20 min with paraformaldehyde (PFA 4% in PBS) at 4°C, permeabilized with 0.1% Triton X-100 in PBS for 20 min, incubated for 1 h with 10% goat serum in PBS as blocking solution, treated with IgG primary antibody against ZO-1 (1:120; Invitrogen), and finally stained with a PBS solution containing goat Alexa Fluor 488-IgG anti-rabbit secondary antibody (1:200; Invitrogen) and Hoechst 33342 (1:1,000; Invitrogen). The staining of the *f*-actin has been carried out with TRITC-conjugated phalloidin (100 μM; Millipore). Confocal fluorescence microscopy was performed by using a laser scanning C2 system (Nikon); 3D reconstruction from *z*-stack acquisitions was obtained by using NIS-Elements software (Nikon).

TEER was measured at day 2, 3, and 4 of co-culture, by using a Millipore Millicell ERS-2 Volt-Ohmmeter (Millipore^TM^). The two electrode tips coated with Ag/AgCl pellet were inserted in the two compartments of the BBB model (upper and lower, corresponding to the luminal and abluminal side of the BBB model, respectively); 700 and 200 μl of medium were respectively placed in the lower and upper compartment before to perform the measurements. Resistance values were then multiplied by the effective CA membrane area (0.33 cm^2^) and expressed as average TEER ± standard deviation (sample size *n* = 6). The TEER of the CA membrane without cells was finally subtracted.

The apparent permeability coefficient *P*_*app*_ of the BBB model has been analyzed. A permeability assay was performed at day 4 by monitoring the crossing of two FITC-dextrans (MW 4 and 70 kDa; Sigma-Aldrich). For these experiments, phenol red-free complete medium was used: 700 μl of medium were added into the bottom compartment (abluminal), and 200 μl of medium in the compartment of the inserts (luminal chamber). The fluorescence of the medium in the abluminal compartment was measured after 4 h of incubation by using a Victor X3 2030 Multilabel Plate Reader (Perkin Elmer^TM^; sample size *n* = 6); 70 μl were collected from the abluminal compartment of each insert for performing the fluorescence measurements and subsequently replaced in the respective compartment. Fluorescence emissions (a.u.) were then converted to FITC-dextran concentrations by using a calibration curve for the 70 kDa (Supplementary Figure 6) and for the 4 kDa (Supplementary Figure 7) FITC-dextran. *P*_*app*_ was calculated following Equation 2, where *dQ/dt* is the cumulative amount of the dextran which has been transported over the membrane, *C*_0_ is the initial concentration of the dextran on the luminal compartment, and *A* is the surface area of the inserts:

(2) $$P_{a\,p\,p}=\frac{d\,Q}{d\,t}\cdot \frac{1}{A\cdot C_{0}}$$

### CA Electrospun Substrates Preparation and Characterization

Solutions of CA (15, 16, 17, and 18% wt) were prepared for electrospinning. A 10 ml syringe (Henke-Sass Wolf) with a 21G 0.8 mm × 50 mm needle (BD Microlance) was loaded with the solutions and connected to a microfluidics syringe pump (NE-1002X, ALA Scientific Instruments). The pump was programmed to dispense a flow rate of 1 ml/h. The electric field was generated by a power supply (Linari Engineering) connected to both the syringe needle and the collector through an applied voltage of 12 kV. A stainless-steel woven wire mesh with 1 mm^2^ holes was positioned at 10 cm distance from the needle tip and was used as support for the electrospun CA fibers. CA solutions with 16, 17, and 18% wt cellulose resulted highly viscous, and electrospinning resulted not possible in these conditions. For this reason, CA electrospun scaffolds were prepared with the 15% wt cellulose solution; three different spinning times (5, 15, and 25 min) were tested and characterized. Optical imaging was carried out with a Zeiss Primotech microscope and SEM imaging was performed as described above. Porosity and fiber size were measured with ImageJ. The optical transmittance of electrospun CA scaffolds (25 min of electrospinning), CA membranes obtained *via* VIPS, and commercially available PET membranes was investigated in the range of 300–800 nm by a spectrophotometer Cary 5000 (Agilent).

### *In vitro* Single-Cell BBB Models on CA Electrospun Substrates

Membranes of commercial inserts have been substituted with the electrospun CA scaffold as described above for the CA membranes obtained *via* VIPS. Electrospun CA scaffolds were a liquid-absorbing material and, conversely to CA-based membranes obtained by VIPS, it was not possible to exploit the double seeding approach to obtain biomimetic multicellular models. Moreover, some of the electrospun substrates started to deteriorate and fray after day 4 in biological medium; this issue did not allow long-term experiments to be performed. For this reason, we planned to set up BBB models by only seeding brain endothelial cells (bEnd.3 cell line, ATCC^®^ CRL-2299TM) on the upper surface of the CA electrospun substrates (seeding density 6 × 10^4^ cells/cm^2^). bEnd.3 cells were selected for electrospun scaffolds, since this brain endothelial cell line was able to better adhere to these porous structures with respect to the hCMEC line. Cell staining, confocal imaging, TEER analysis, and the analysis of dextran permeability were conducted as described for CA membranes obtained *via* VIPS.

## Data Availability Statement

The raw data supporting the conclusions of this article will be made available by the authors, without undue reservation.

## Author Contributions

EM, AM, and GC conceived the experiments. AM and MB performed the *in vitro* experiments. MB and UB obtained the scaffolds. AM and MB carried out the statistical analysis. AM and GC wrote the manuscript. All authors contributed to the article and approved the submitted version.

## Conflict of Interest

The authors declare that the research was conducted in the absence of any commercial or financial relationships that could be construed as a potential conflict of interest.

## Footnotes

1 https://imagej.nih.gov/ij/
