# Supplementary material for: Porous Optically Transparent Cellulose Acetate Scaffolds for Biomimetic Blood-Brain Barrierin vitro Models
Source: Front Bioeng Biotechnol. 2021 Feb 10;9:630063. doi: 10.3389/fbioe.2021.630063 (PMC7928328; doi:10.3389/fbioe.2021.630063)
Supplement: Supplementary file 1 [file Data_Sheet_1.docx]

Supplementary Material

**Porous optically transparent cellulose acetate scaffolds for biomimetic blood-brain barrier *in vitro* models**

Attilio Marino^1,†,*^, Micol Baronio^2,†^, Umberto Buratti^2^, Elisa Mele^3,*^, Gianni Ciofani^1,*^

^1^Smart Bio-Interfaces, Istituto Italiano di Tecnologia, Viale Rinaldo Piaggio 34, 56025 Pontedera, Italy

^2^Department of Mechanical and Aerospace Engineering, Politecnico di Torino, Corso Duca degli Abruzzi 24, 10129 Torino, Italy

^3^Materials Department, Loughborough University, Epinal Way, LE11 3TU Loughborough, UK

^†^These authors equally contributed to this work

*** Correspondence:***attilio.marino@iit.it; *gianni.ciofani@iit.it; *e.mele2@lboro.ac.uk

a)

b)

c)

**Figure S1.** Photos of representative membranes produced by VIPS with a) 15.0%, b) 10.0%, and c) 7.5% wt CA solutions.


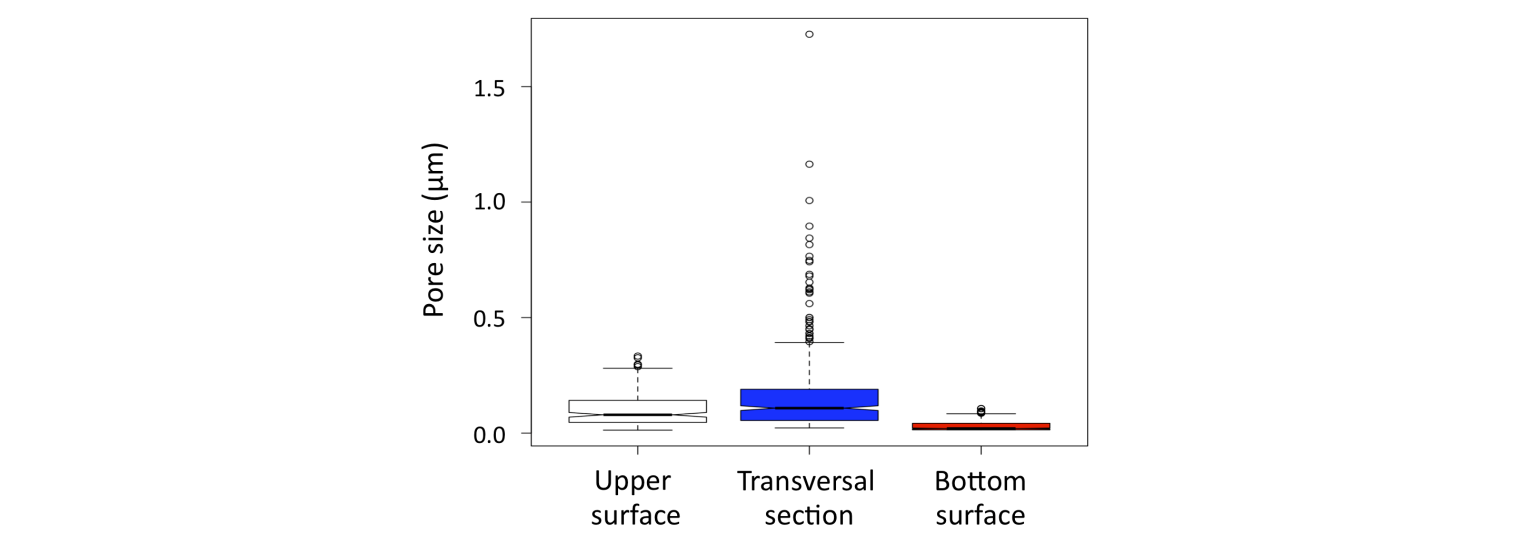
**Figure S2.** Pore size distributions with outliers of upper, bottom and transversal surfaces of the CA membranes fabricated by using 25% H_2_O + 75% EtOH non-solvent.





**Figure S3.** Optical transmittance of CA membranes obtained *via* VIPS (black), electrospun CA scaffolds (blue), and commercially available poly(ethylene terephthalate) (PET) membranes.


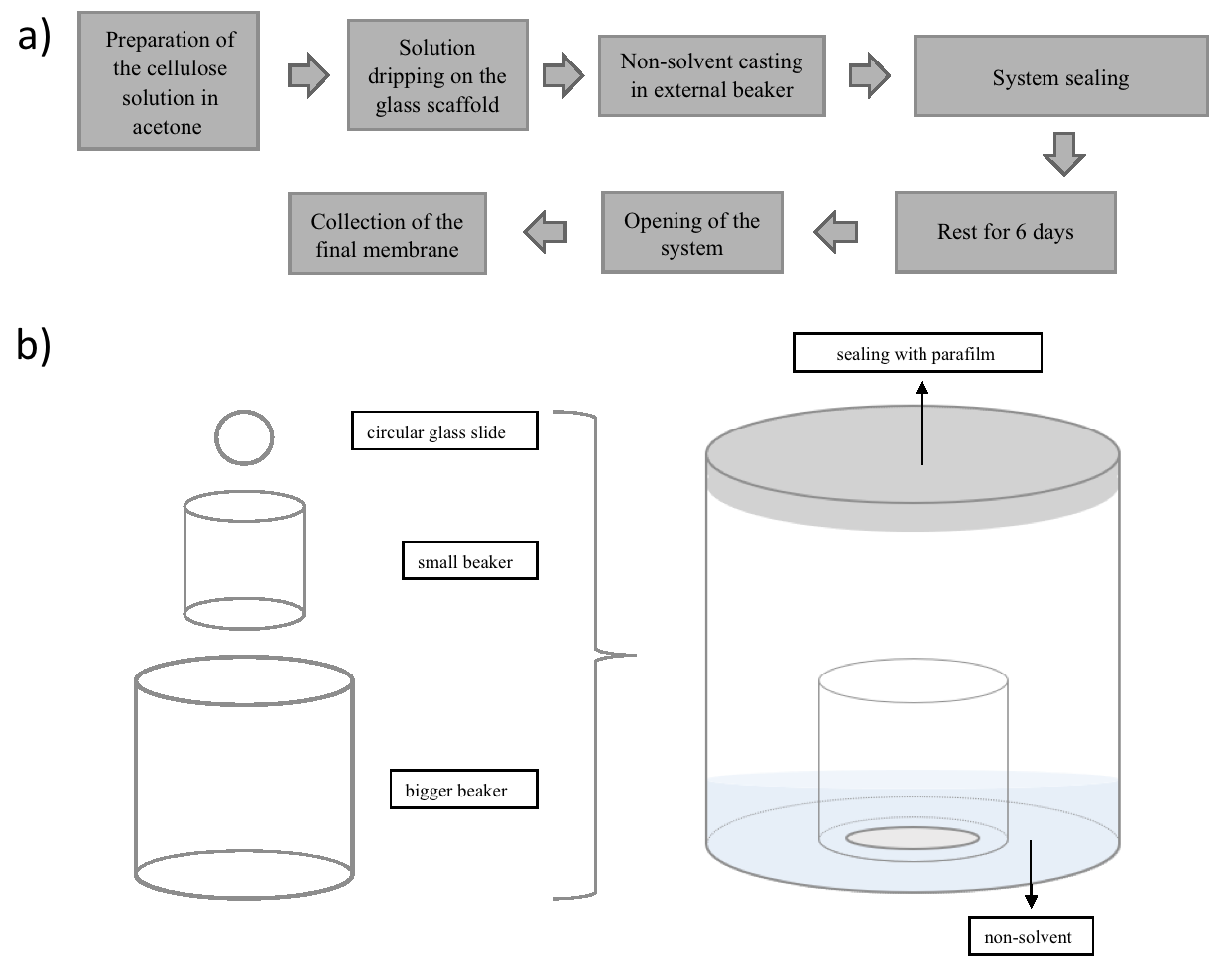
**Figure S4.** a) Flowchart and b) scheme of the experimental procedure for the fabrication of cellulose acetate membranes by VIPS.


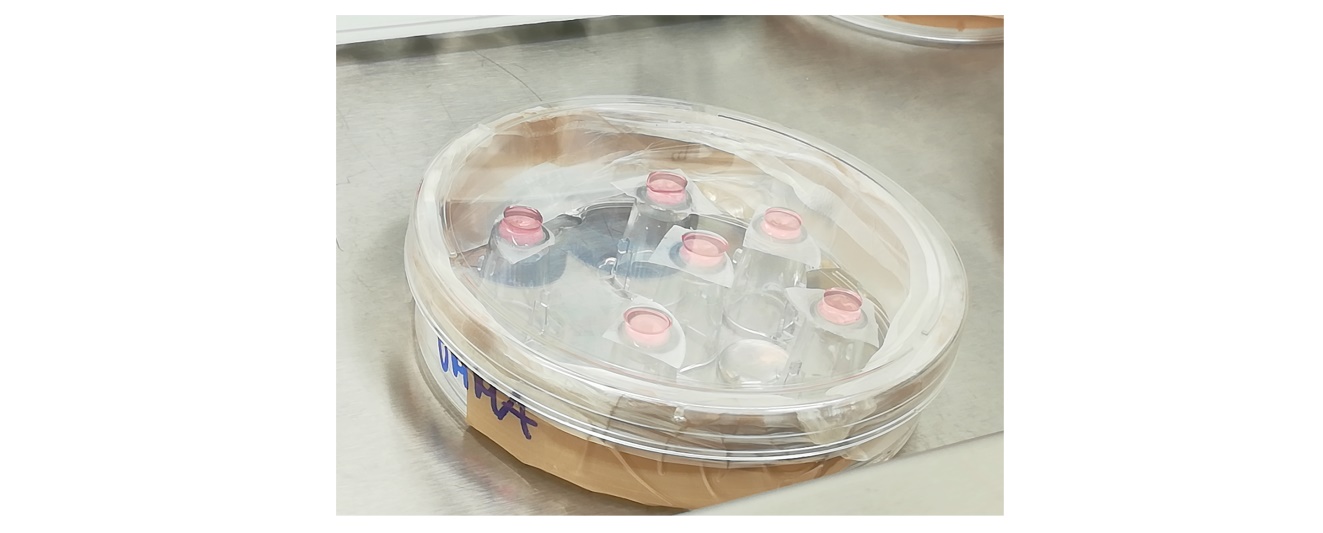
**Figure S5.** Astrocyte seeding on the bottom surface of CA membranes obtained *via* VIPS. The surface of a Petri dish is placed on the inserts, in direct contact with the drop of medium used for astrocyte seeding in order to avoid medium leaking through the membranes, by exploiting surface tension.


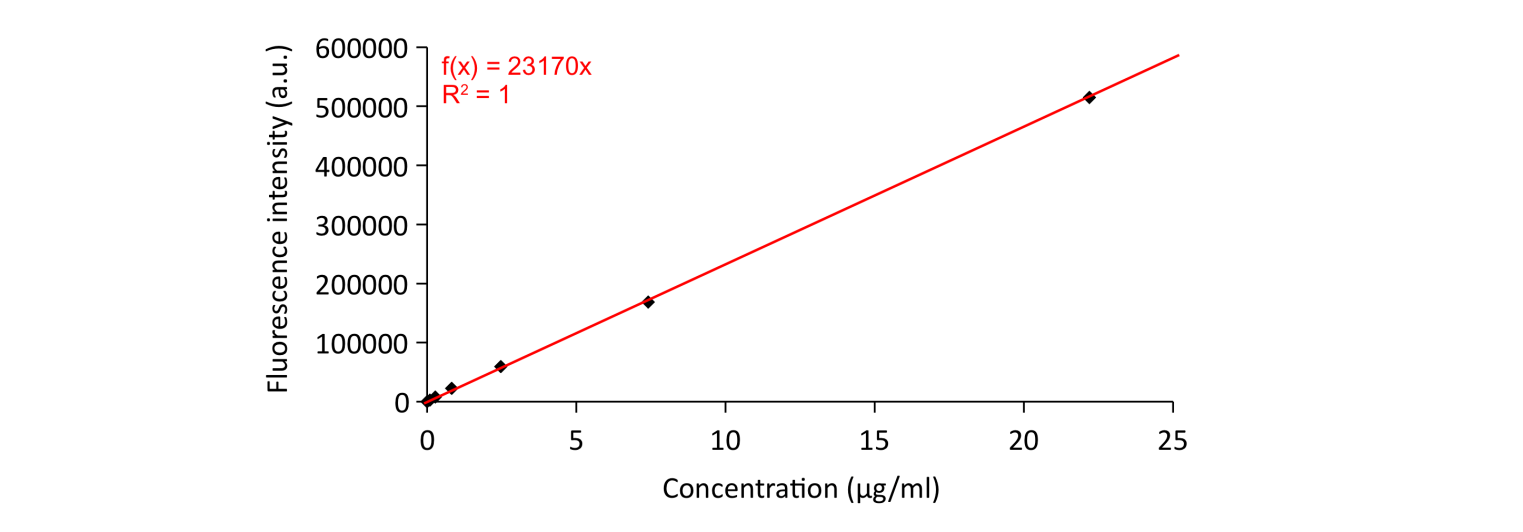
**Figure S6.** Calibration curve showing the fluorescence emissions (a.u.) of different concentrations of 70 kDa FITC-dextran.


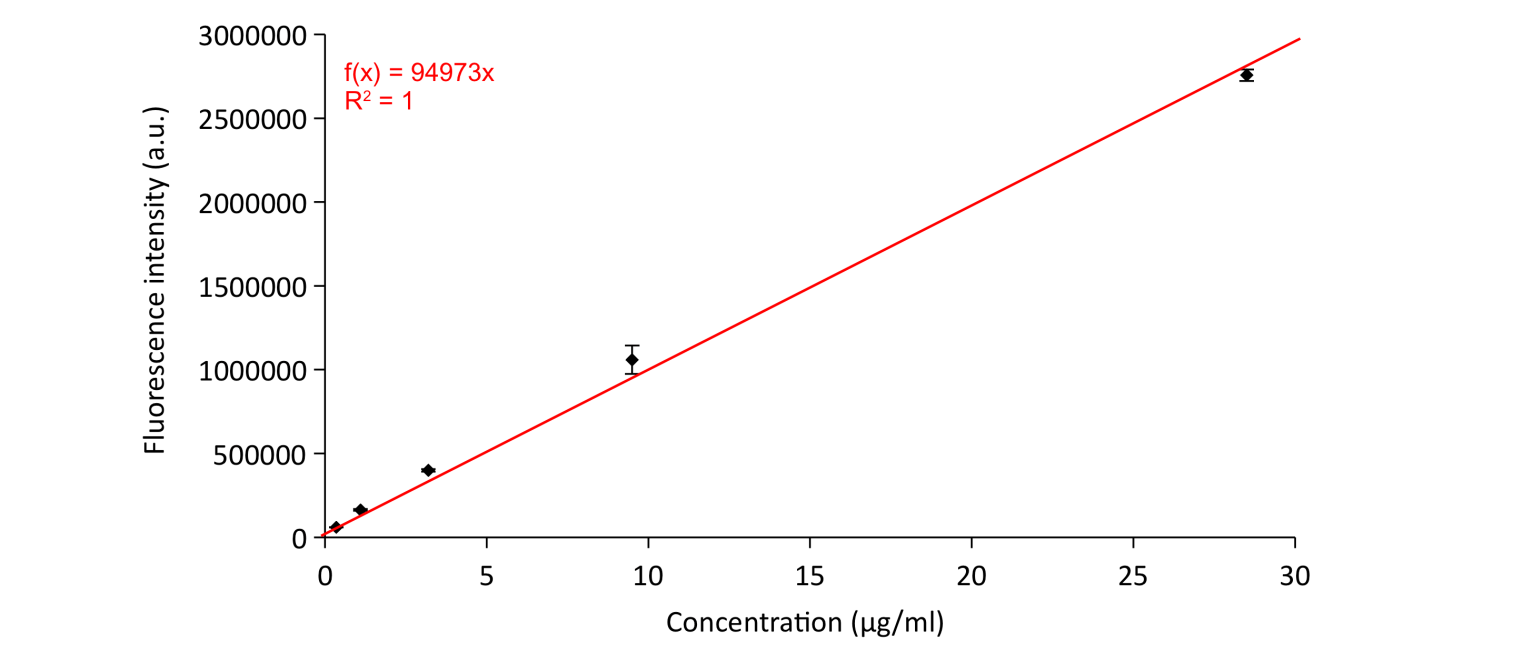
**Figure S7.** Calibration curve showing the fluorescence emissions (a.u.) of different concentrations of 4 kDa FITC-dextran.
